# Supplementary material for: A fault-intrusion-tolerant system and deadline-aware algorithm for scheduling scientific workflow in the cloud
Source: PeerJ Comput Sci. 2021 Nov 2;7:e747. doi: 10.7717/peerj-cs.747 (PMC8576554; doi:10.7717/peerj-cs.747)
Supplement: Supplemental Information 6 [file peerj-cs-07-747-s006.zip › Myproject1.1/docs/org/cloudbus/cloudsim/class-use/Host.html]

Uses of Class org.cloudbus.cloudsim.Host


JavaScript is disabled on your browser.


- Overview
- Package
- Class
- Use
- Tree
- Deprecated
- Index
- Help

- Prev
- Next

- Frames
- No Frames

- All Classes

## Uses of Class org.cloudbus.cloudsim.Host

- Packages that use Host

  | Package | Description |
  | --- | --- |
  |  |  |
  | --- | --- |
  | org.cloudbus.cloudsim |  |
  | org.cloudbus.cloudsim.examples.power |  |
  | org.cloudbus.cloudsim.lists |  |
  | org.cloudbus.cloudsim.network.datacenter |  |
  | org.cloudbus.cloudsim.power |  |
- - ### Uses of Host in org.cloudbus.cloudsim

    Subclasses of Host in org.cloudbus.cloudsim

    | Modifier and Type | Class and Description |
    | --- | --- |
    |  |  |
    | --- | --- |
    | `class` | `HostDynamicWorkload` The class of a host supporting dynamic workloads and performance degradation. |

    Methods in org.cloudbus.cloudsim with type parameters of type Host

    | Modifier and Type | Method and Description |
    | --- | --- |
    |  |  |
    | --- | --- |
    | `<T extends Host>  java.util.List<T>` | Datacenter.`getHostList()` Gets the host list. |
    | `<T extends Host>  java.util.List<T>` | DatacenterCharacteristics.`getHostList()` Gets the host list. |
    | `<T extends Host>  java.util.List<T>` | VmAllocationPolicy.`getHostList()` Gets the host list. |
    | `protected <T extends Host>  void` | DatacenterCharacteristics.`setHostList(java.util.List<T> hostList)` Sets the host list. |

    Methods in org.cloudbus.cloudsim that return Host

    | Modifier and Type | Method and Description |
    | --- | --- |
    |  |  |
    | --- | --- |
    | `Host` | Vm.`getHost()` Gets the host. |
    | `abstract Host` | VmAllocationPolicy.`getHost(int vmId, int userId)` Get the host that is executing the given VM belonging to the given user. |
    | `Host` | VmAllocationPolicySimple.`getHost(int vmId, int userId)` Gets the host that is executing the given VM belonging to the given user. |
    | `abstract Host` | VmAllocationPolicy.`getHost(Vm vm)` Get the host that is executing the given VM belonging to the given user. |
    | `Host` | VmAllocationPolicySimple.`getHost(Vm vm)` Gets the host that is executing the given VM belonging to the given user. |
    | `Host` | DatacenterCharacteristics.`getHostWithFreePe()` Gets a Machine with at least one empty Pe. |
    | `Host` | DatacenterCharacteristics.`getHostWithFreePe(int peNumber)` Gets a Machine with at least a given number of free Pe. |

    Methods in org.cloudbus.cloudsim that return types with arguments of type Host

    | Modifier and Type | Method and Description |
    | --- | --- |
    |  |  |
    | --- | --- |
    | `java.util.Map<java.lang.String,Host>` | VmAllocationPolicySimple.`getVmTable()` Gets the vm table. |

    Methods in org.cloudbus.cloudsim with parameters of type Host

    | Modifier and Type | Method and Description |
    | --- | --- |
    |  |  |
    | --- | --- |
    | `abstract boolean` | VmAllocationPolicy.`allocateHostForVm(Vm vm, Host host)` Allocates a specified host for a given VM. |
    | `boolean` | VmAllocationPolicySimple.`allocateHostForVm(Vm vm, Host host)` |
    | `void` | Vm.`setHost(Host host)` Sets the host that runs this VM. |

    Method parameters in org.cloudbus.cloudsim with type arguments of type Host

    | Modifier and Type | Method and Description |
    | --- | --- |
    |  |  |
    | --- | --- |
    | `protected void` | VmAllocationPolicy.`setHostList(java.util.List<? extends Host> hostList)` Sets the host list. |
    | `protected void` | VmAllocationPolicySimple.`setVmTable(java.util.Map<java.lang.String,Host> vmTable)` Sets the vm table. |

    Constructor parameters in org.cloudbus.cloudsim with type arguments of type Host

    Constructor and Description

    `DatacenterCharacteristics(java.lang.String architecture,
    java.lang.String os,
    java.lang.String vmm,
    java.util.List<? extends Host> hostList,
    double timeZone,
    double costPerSec,
    double costPerMem,
    double costPerStorage,
    double costPerBw)`

    Allocates a new DatacenterCharacteristics object.

    `VmAllocationPolicy(java.util.List<? extends Host> list)`

    Allocates a new VmAllocationPolicy object.

    `VmAllocationPolicySimple(java.util.List<? extends Host> list)`

    Creates the new VmAllocationPolicySimple object.
  - ### Uses of Host in org.cloudbus.cloudsim.examples.power

    Method parameters in org.cloudbus.cloudsim.examples.power with type arguments of type Host

    | Modifier and Type | Method and Description |
    | --- | --- |
    |  |  |
    | --- | --- |
    | `protected static double` | Helper.`getSlaTimePerActiveHost(java.util.List<Host> hosts)` Gets the sla time per active host. |
    | `protected static double` | Helper.`getSlaTimePerHost(java.util.List<Host> hosts)` Gets the sla time per host. |
    | `static java.util.List<java.lang.Double>` | Helper.`getTimesBeforeHostShutdown(java.util.List<Host> hosts)` Gets the times before host shutdown. |
    | `static void` | Helper.`printMetricHistory(java.util.List<? extends Host> hosts, PowerVmAllocationPolicyMigrationAbstract vmAllocationPolicy)` Prints the metric history. |
    | `static void` | Helper.`writeMetricHistory(java.util.List<? extends Host> hosts, PowerVmAllocationPolicyMigrationAbstract vmAllocationPolicy, java.lang.String outputPath)` Write metric history. |
  - ### Uses of Host in org.cloudbus.cloudsim.lists

    Methods in org.cloudbus.cloudsim.lists with type parameters of type Host

    | Modifier and Type | Method and Description |
    | --- | --- |
    |  |  |
    | --- | --- |
    | `static <T extends Host>  T` | HostList.`getById(java.util.List<T> hostList, int id)` Gets the Machine object for a particular ID. |
    | `static <T extends Host>  T` | HostList.`getHostWithFreePe(java.util.List<T> hostList)` Gets a Machine with free Pe. |
    | `static <T extends Host>  T` | HostList.`getHostWithFreePe(java.util.List<T> hostList, int pesNumber)` Gets a Machine with a specified number of free Pe. |
    | `static <T extends Host>  int` | HostList.`getNumberOfBusyPes(java.util.List<T> hostList)` Gets the total number of BUSY PEs for all Machines. |
    | `static <T extends Host>  int` | HostList.`getNumberOfFreePes(java.util.List<T> hostList)` Gets the total number of FREE or non-busy PEs for all Machines. |
    | `static <T extends Host>  int` | HostList.`getNumberOfPes(java.util.List<T> hostList)` Gets the total number of PEs for all Machines. |
    | `static <T extends Host>  boolean` | HostList.`setPeStatus(java.util.List<T> hostList, int status, int hostId, int peId)` Sets the particular Pe status on a Machine. |
  - ### Uses of Host in org.cloudbus.cloudsim.network.datacenter

    Subclasses of Host in org.cloudbus.cloudsim.network.datacenter

    | Modifier and Type | Class and Description |
    | --- | --- |
    |  |  |
    | --- | --- |
    | `class` | `NetworkHost` NetworkHost class extends Host to support simulation of networked datacenters. |

    Methods in org.cloudbus.cloudsim.network.datacenter that return Host

    | Modifier and Type | Method and Description |
    | --- | --- |
    |  |  |
    | --- | --- |
    | `Host` | NetworkVmAllocationPolicy.`getHost(int vmId, int userId)` Gets the host that is executing the given VM belonging to the given user. |
    | `Host` | NetworkVmAllocationPolicy.`getHost(Vm vm)` Gets the host that is executing the given VM belonging to the given user. |

    Methods in org.cloudbus.cloudsim.network.datacenter that return types with arguments of type Host

    | Modifier and Type | Method and Description |
    | --- | --- |
    |  |  |
    | --- | --- |
    | `java.util.Map<java.lang.String,Host>` | NetworkVmAllocationPolicy.`getVmTable()` Gets the vm table. |

    Methods in org.cloudbus.cloudsim.network.datacenter with parameters of type Host

    | Modifier and Type | Method and Description |
    | --- | --- |
    |  |  |
    | --- | --- |
    | `boolean` | NetworkVmAllocationPolicy.`allocateHostForVm(Vm vm, Host host)` |

    Method parameters in org.cloudbus.cloudsim.network.datacenter with type arguments of type Host

    | Modifier and Type | Method and Description |
    | --- | --- |
    |  |  |
    | --- | --- |
    | `protected void` | NetworkVmAllocationPolicy.`setVmTable(java.util.Map<java.lang.String,Host> vmTable)` Sets the vm table. |

    Constructor parameters in org.cloudbus.cloudsim.network.datacenter with type arguments of type Host

    Constructor and Description

    `NetworkVmAllocationPolicy(java.util.List<? extends Host> list)`

    Creates the new VmAllocationPolicySimple object.
  - ### Uses of Host in org.cloudbus.cloudsim.power

    Subclasses of Host in org.cloudbus.cloudsim.power

    | Modifier and Type | Class and Description |
    | --- | --- |
    |  |  |
    | --- | --- |
    | `class` | `PowerHost` PowerHost class enables simulation of power-aware hosts. |
    | `class` | `PowerHostUtilizationHistory` The class of a host that stores its CPU utilization history. |

    Methods in org.cloudbus.cloudsim.power that return Host

    | Modifier and Type | Method and Description |
    | --- | --- |
    |  |  |
    | --- | --- |
    | `Host` | PowerVmAllocationPolicyAbstract.`getHost(int vmId, int userId)` |
    | `Host` | PowerVmAllocationPolicyAbstract.`getHost(Vm vm)` |

    Methods in org.cloudbus.cloudsim.power that return types with arguments of type Host

    | Modifier and Type | Method and Description |
    | --- | --- |
    |  |  |
    | --- | --- |
    | `java.util.Map<java.lang.String,Host>` | PowerVmAllocationPolicyAbstract.`getVmTable()` Gets the vm table. |

    Methods in org.cloudbus.cloudsim.power with parameters of type Host

    | Modifier and Type | Method and Description |
    | --- | --- |
    |  |  |
    | --- | --- |
    | `boolean` | PowerVmAllocationPolicyAbstract.`allocateHostForVm(Vm vm, Host host)` |

    Method parameters in org.cloudbus.cloudsim.power with type arguments of type Host

    | Modifier and Type | Method and Description |
    | --- | --- |
    |  |  |
    | --- | --- |
    | `PowerHost` | PowerVmAllocationPolicyMigrationAbstract.`findHostForVm(Vm vm, java.util.Set<? extends Host> excludedHosts)` Find host for vm. |
    | `protected java.util.List<java.util.Map<java.lang.String,java.lang.Object>>` | PowerVmAllocationPolicyMigrationAbstract.`getNewVmPlacement(java.util.List<? extends Vm> vmsToMigrate, java.util.Set<? extends Host> excludedHosts)` Gets the new vm placement. |
    | `protected java.util.List<java.util.Map<java.lang.String,java.lang.Object>>` | PowerVmAllocationPolicyMigrationAbstract.`getNewVmPlacementFromUnderUtilizedHost(java.util.List<? extends Vm> vmsToMigrate, java.util.Set<? extends Host> excludedHosts)` Gets the new vm placement from under utilized host. |
    | `protected PowerHost` | PowerVmAllocationPolicyMigrationAbstract.`getUnderUtilizedHost(java.util.Set<? extends Host> excludedHosts)` Gets the under utilized host. |

    Constructor parameters in org.cloudbus.cloudsim.power with type arguments of type Host

    Constructor and Description

    `PowerVmAllocationPolicyAbstract(java.util.List<? extends Host> list)`

    Instantiates a new power vm allocation policy abstract.

    `PowerVmAllocationPolicyMigrationAbstract(java.util.List<? extends Host> hostList,
    PowerVmSelectionPolicy vmSelectionPolicy)`

    Instantiates a new power vm allocation policy migration abstract.

    `PowerVmAllocationPolicyMigrationInterQuartileRange(java.util.List<? extends Host> hostList,
    PowerVmSelectionPolicy vmSelectionPolicy,
    double safetyParameter,
    PowerVmAllocationPolicyMigrationAbstract fallbackVmAllocationPolicy)`

    Instantiates a new power vm allocation policy migration mad.

    `PowerVmAllocationPolicyMigrationInterQuartileRange(java.util.List<? extends Host> hostList,
    PowerVmSelectionPolicy vmSelectionPolicy,
    double safetyParameter,
    PowerVmAllocationPolicyMigrationAbstract fallbackVmAllocationPolicy,
    double utilizationThreshold)`

    Instantiates a new power vm allocation policy migration mad.

    `PowerVmAllocationPolicyMigrationLocalRegression(java.util.List<? extends Host> hostList,
    PowerVmSelectionPolicy vmSelectionPolicy,
    double safetyParameter,
    double schedulingInterval,
    PowerVmAllocationPolicyMigrationAbstract fallbackVmAllocationPolicy)`

    Instantiates a new power vm allocation policy migration local regression.

    `PowerVmAllocationPolicyMigrationLocalRegression(java.util.List<? extends Host> hostList,
    PowerVmSelectionPolicy vmSelectionPolicy,
    double safetyParameter,
    double schedulingInterval,
    PowerVmAllocationPolicyMigrationAbstract fallbackVmAllocationPolicy,
    double utilizationThreshold)`

    Instantiates a new power vm allocation policy migration local regression.

    `PowerVmAllocationPolicyMigrationLocalRegressionRobust(java.util.List<? extends Host> hostList,
    PowerVmSelectionPolicy vmSelectionPolicy,
    double safetyParameter,
    double schedulingInterval,
    PowerVmAllocationPolicyMigrationAbstract fallbackVmAllocationPolicy)`

    Instantiates a new power vm allocation policy migration local regression.

    `PowerVmAllocationPolicyMigrationLocalRegressionRobust(java.util.List<? extends Host> hostList,
    PowerVmSelectionPolicy vmSelectionPolicy,
    double safetyParameter,
    double schedulingInterval,
    PowerVmAllocationPolicyMigrationAbstract fallbackVmAllocationPolicy,
    double utilizationThreshold)`

    Instantiates a new power vm allocation policy migration local regression.

    `PowerVmAllocationPolicyMigrationMedianAbsoluteDeviation(java.util.List<? extends Host> hostList,
    PowerVmSelectionPolicy vmSelectionPolicy,
    double safetyParameter,
    PowerVmAllocationPolicyMigrationAbstract fallbackVmAllocationPolicy)`

    Instantiates a new power vm allocation policy migration mad.

    `PowerVmAllocationPolicyMigrationMedianAbsoluteDeviation(java.util.List<? extends Host> hostList,
    PowerVmSelectionPolicy vmSelectionPolicy,
    double safetyParameter,
    PowerVmAllocationPolicyMigrationAbstract fallbackVmAllocationPolicy,
    double utilizationThreshold)`

    Instantiates a new power vm allocation policy migration mad.

    `PowerVmAllocationPolicyMigrationStaticThreshold(java.util.List<? extends Host> hostList,
    PowerVmSelectionPolicy vmSelectionPolicy,
    double utilizationThreshold)`

    Instantiates a new power vm allocation policy migration mad.

    `PowerVmAllocationPolicySimple(java.util.List<? extends Host> list)`

    Instantiates a new power vm allocation policy simple.

- Overview
- Package
- Class
- Use
- Tree
- Deprecated
- Index
- Help

- Prev
- Next

- Frames
- No Frames

- All Classes
